# Enhancement of Phonological Memory Following Transcranial Magnetic Stimulation (TMS)

**DOI:** 10.1155/2006/469132

**Published:** 2006-11-27

**Authors:** Matthew P. Kirschen, Mathew S. Davis-Ratner, Thomas E. Jerde, Pam Schraedley-Desmond, John E. Desmond

**Affiliations:** ^1^Department of RadiologyStanford UniversityStanfordCA 94305USA; ^2^Neurosciences ProgramStanford UniversityStanfordCA 94305USA; ^3^Department of NeurologyJohns Hopkins UniversityBaltimoreMD 21205USA

**Keywords:** Transcranial magnetic stimulation (TMS), verbal working memory (VWM), cognitive enhancement, neuroimaging, functional magnetic resonance imaging (fMRI), phonological memory

## Abstract

Phonologically similar items (mell, rell, gell) are more difficult to remember than dissimilar items (shen, floy, stap), likely because of mutual interference of the items in the phonological store. Low-frequency transcranial magnetic stimulation (TMS), guided by functional magnetic resonance imaging (fMRI) was used to disrupt this phonological confusion by stimulation of the left inferior parietal (LIP) lobule. Subjects received TMS or placebo stimulation while remembering sets of phonologically similar or dissimilar pseudo-words. Consistent with behavioral performance of patients with neurological damage, memory for phonologically similar, but not dissimilar, items was enhanced following TMS relative to placebo stimulation. Stimulation of a control region of the brain did not produce any changes in memory performance. These results provide new insights into how the brain processes verbal information by establishing the necessity of the inferior parietal region for optimal phonological storage. A mechanism is proposed for how TMS reduces phonological confusion and leads to facilitation of phonological memory.

## 1. Introduction

Transcranial magnetic stimulation (TMS) has recently emerged as a complementary modality to functional neuroimaging for the study of human cognition. Using a powerful rapidly changing current through a coil placed on the scalp [32], the application of TMS can selectively disrupt normal neural signaling in specific regions, revealing neural structures necessary for certain cognitive functions. By contrast, patient lesion studies, while valuable for determining brain-behavior relationships, are confounded by the presence of compensatory mechanisms and functional rewiring that might obscure the original function of the damaged tissue. TMS has been shown to disrupt working memory performance [8,10,15,16,23–25], and paradoxically facilitate memory performance [19,29], but to date no studies have demonstrated whether TMS can reproduce the neurobehavioral pattern of VWM similar to that observed in LIP-damaged patients.

Baddeley [2,4] proposed a mechanism for VWM, referred to as the phonological loop, which consists of two components: a phonological short-term store, which can hold speech-related information for 1–2 seconds, and an articulatory control system, which serves sub-vocally to refresh the contents of the phonological store. Behavioral evidence [21], neuroimaging studies [1,6,12,17,18,20,27] and patient reports [30,31],

ISSN 0953-4180/06/$17.00 © 2006 – IOS Press and the authors. All rights reserved suggest that the phonological store and the articulatory control system are independent processes and have distinct neuroanatomical correlates. The phonological store has been functionally linked with the left inferior parietal (LIP) region and the articulatory control system with the left inferior frontal region (LIF). Furthermore, the LIP region is primarily activated during memory rehearsal, while the LIF region is active during both memory encoding and rehearsal [5]. The integrity of the phonological store is classically evaluated through a behavioral phenomenon called the phonological similarity effect (PSE) – the immediate recall for a list of phonologically dissimilar stimuli (e.g., *J, M, R, K, S, L*) is greater than recall for a list of phonologically similar stimuli (e.g., *V, B, T, C, P, G*). The PSE is thought to arise because of interference between items in the short-term phonological store; thus, the absence of this effect is thought to reflect a deficit in the phonological store [7] and by extension, a compromise in the LIP region.

Using functional magnetic resonance imaging (fMRI) to guide TMS localization, we transiently disrupted neuronal processing in the LIP region of normal human subjects. We applied low-frequency TMS to the LIP region identified by subject-specific fMRI activation while each subject performed a modified VWM task to examine the PSE. We hypothesized that TMS to the LIP would maximally alter VWM performance on trials with high demand on the phonological store. In the present experiment, such demand would be most pronounced when subjects studied and rehearsed a phonologically similar list of stimuli.

## 2. Methods

### *2.1. Subjects*

Subjects were 30 right-handed subjects (15 male, 15 female) who participated for monetary compensation. Subjects were native speakers of English and on average 22.0 $\pm$ 2.5 years old ($\pm$ SD). Informed consent was obtained from each individual subject prior to their participation in the experiment which was approved by the Institutional Review Board at Stanford University.

### *2.2. Functional MRI behavioral task*

To guide targeting of TMS, subjects performed a verbal working memory task similar to those used in previous investigations from our laboratory [5,6,9,20]. Subjects were instructed to remember 2 (low memory load) or 6 (high memory load) randomly generated consonants presented at a rate of 2 items per second in uppercase font. Sequential presentation was used to ensure subjects were properly encoding the items rather than remembering their placement or orientation in an array. Subjects rehearsed these letters sub-vocally during a 6000 ms retention interval and were instructed not to use mnemonic or other non-phonological memory strategies. A lowercase probe letter was then presented and subjects indicated with a button press if this probe letter matched a remembered letter in the preceding list (yes – right index finger; no – right middle finger). The probe item was present for the initial 1500 ms of a 2000 ms response interval, followed by an inter-trial-interval (ITI) of 3000 ms. Responses to the probe item were not accepted during the ITI and a failure to make a response did not inhibit the start of the subsequent trial. The probe letter matched one of the target letters on half the trials and the position of the matching target letter in the presented sequence was counterbalanced over all possible positions. The order in which the subjects received the different list lengths was also counterbalanced. A focus mark presented for 750 ms (followed by a 250 ms delay) indicated the start of each trial. Subjects received approximately 15 trials of practice prior to beginning the experiment. Subjects were instructed to be fast and accurate in their responses. Each subject completed a total of 48 trials presented in a block design format with two trials per block.

Subjects also completed a finger tapping task that was used to localize motor cortex and determine TMS threshold. When cued, subjects repeatedly tapped the thumb of the right hand with each of the other 4 digits individually for 30 seconds. This was followed by 30 seconds of rest. This cycle was repeated for 5 minutes.

### *2.3. MRI data acquisition and analysis*

MRI data were acquired on a General Electric Signa 1.5T whole body scanner. A three-dimensional T1-weighted spoiled gradient echo (SPGR) scan was collected for anatomical coregistration with the functional volumes. Whole-brain fMRI was performed with a single-interleave T2*-weighted gradient echo spiral in/out pulse sequence [14] (in-plane resolution = 3.75 mm, 6 mm slice thickness, TR = 2 seconds). Following timing and motion correction, subject activations were assessed using the general linear model method of SPM2 software (Wellcome Department of Cognitive Neurology) [13]. The LIP activation for each

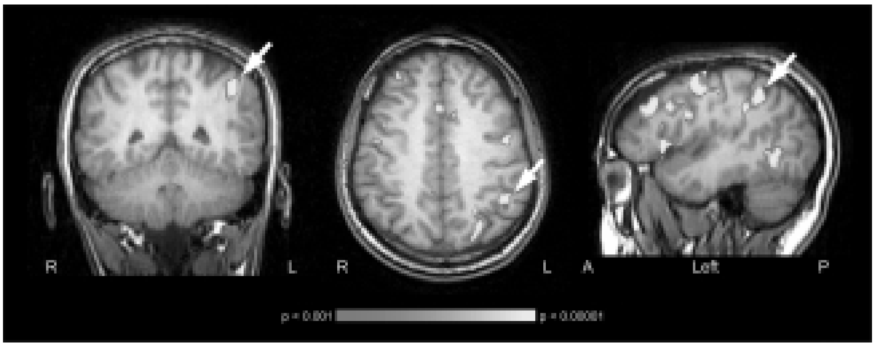

Fig. 1. Functional MRI activation maps, Activation maps are displayed in 3 planes (left to right: coronal, axial, sagittal) indicating VWM activation in the LIP lobule (white arrows) from a representative subject. Activations are displayed on high resolution T1-weighted 3-dimensional SPGR images.

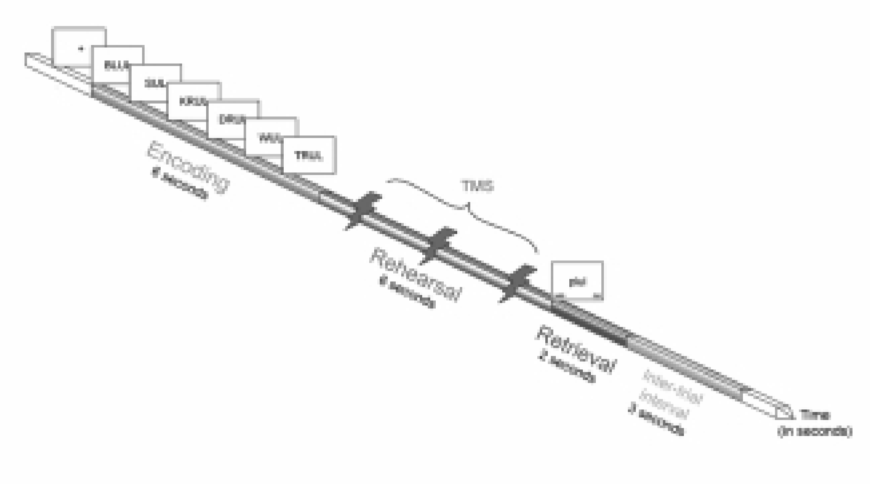

Fig. 2. Schematic of a TMS trial, The Sternberg VWM paradigm [28] has three phases: encoding, maintenance and retrieval. During the encoding phase, subjects studied a list of 6 short phonologically similar (depicted here) or phonologically dissimilar pseudo-words presented at a rate of 1 item per second. During the rehearsal interval, three TMS stimulations were applied at 1, 3, and 5 seconds following the last stimulus. A probe item was then visually presented during the retrieval phase, followed by an inter-trial-interval. A focus mark indicated the start of each trial.

subject was defined using the memory load 6 versus memory load 2 contrast (Fig. 1). If several activations were visible in the LIP region, the one closest to the mean LIP activation during rehearsal, as determined by Chen and Desmond [5], was chosen.

### 2.4. TMS behavioral task

Subjects were instructed to remember a list of 6 short pseudo-words taken from a phonological similarity database created by the authors (Fig. 2). The stimuli were visually presented at 1 item per second in uppercase font. Subjects verbally repeated each word after its presentation to ensure phonological encoding and prevent the use of alternative memory strategies. Subjects rehearsed these stimuli sub-vocally during a 6 second rehearsal interval. A lowercase pseudo-word was then visually presented and subjects indicated with a button press if this probe stimulus matched a remembered stimulus in the preceding list. Both accuracy and response time were measured for each response.

Half of the lists of pseudo-word study items contained stimuli that were phonologically similar while the other half contained phonologically dissimilar items. For each set of lists, an equal number of probe and lure test items were used. For the phonologically similar lists, a lure was phonologically similar to the stimuli, but not presented on the list. The position of the probe/lure was counterbalanced over all presentation positions. Table 1 summarizes the study and test item conditions that were presented to the subjects. Each subject completed 5 experimental sessions:

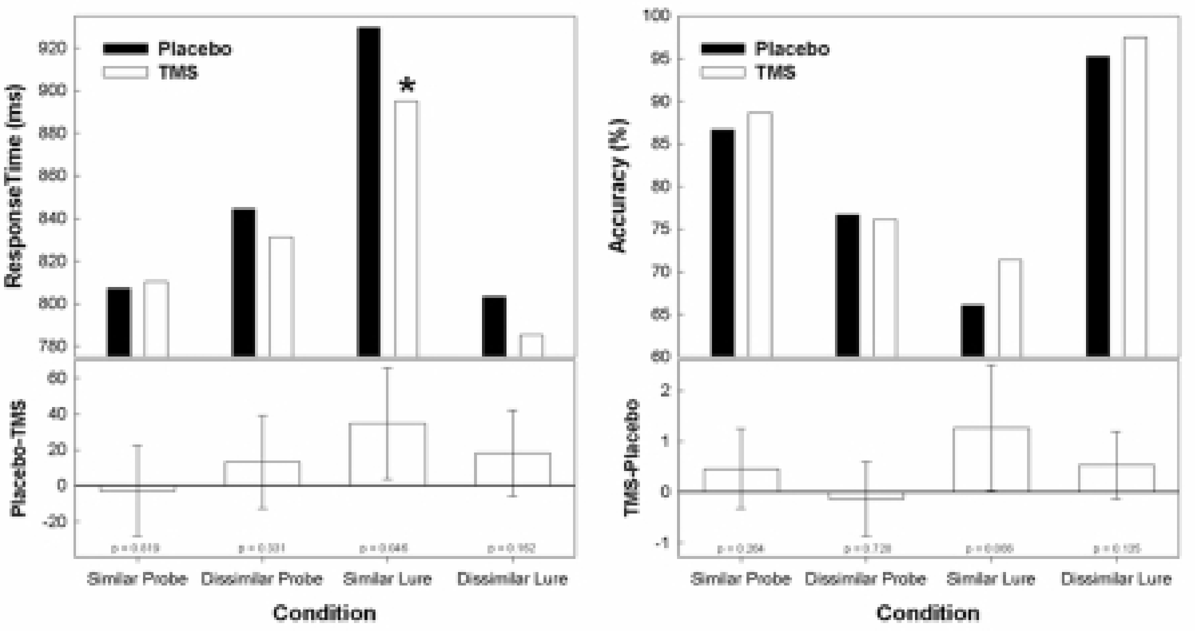

Fig. 3. Response time and accuracy plots for LIP stimulation, The left plot shows a graph of mean response time for TMS (white bars) and placebo (black bars) stimulation for the 4 behavioral conditions – (a) phonologically similar probes, (b) phonologically dissimilar probes, (c) phonologically similar lures, and (d) phonologically dissimilar lures. The lower portion of the plot shows the latency difference between TMS and placebo simulation plotted with 95% confidence intervals. Results from two-tailed paired samples t-tests are indicated for each condition. Average response times were computed based on correct trials only. An asterisk denotes conditions with significant behavioral differences between TMS and placebo stimulation (as visualized by confidence intervals that do not include zero). The right plot displays analogous graphs for the accuracy data.

1 practice, 2 with TMS, and 2 with placebo simulation. Session order was counterbalanced between two orders: TMS-placebo-TMS-placebo and TMS-placebo-placebo-TMS. Each session was comprised of 48 trials, 24 phonologically similar lists and 24 phonologically dissimilar lists, and took approximately 14.5 minutes to complete. The order of the trials within each session was randomized. Half of the subjects ($N = 15$) performed the PSE task with TMS and placebo stimulation to the LIP, while the remaining subjects ($N = 15$) had TMS and placebo stimulation applied to a control region, the parieto-occipital junction at the midline. Additionally, a subset of subjects in the LIP group participated in an additional 2 baseline sessions with neither TMS nor placebo stimulation. The TMS experiment occurred within one week of the fMRI scanning session.

### 2.5. *Phonological similarity database*

A comprehensive phonetic alphabet of consonants (i.e., 'b', 'br', 'bl', 'c') was created to serve as the beginning of each short pseudo-word. A systematic approach was then applied to find vowel-based endings that worked with each consonant beginning (e.g., 'ab', 'abe', 'ack', 'act', 'ad'). Through a combination of consonant beginning and vowel-based endings, a database was created of $>$6000 pseudo-words in phonologically similar lists. Items were eliminated if they were found in an English dictionary, phonetic duplicates, difficult pronunciations, contained inappropriate or slang language, or if an item did not phonetically sound like other words with the same ending. After all exclusions, the database contained 5529 pseudo-words with 207 different phonetic constructions.

To create a set of phonologically similar study items, one vowel-based ending was selected randomly from the database, and a random set of pseudo-words was drawn from that ending's list. For phonologically dissimilar study items, a unique set of vowel-based endings was randomly selected from the database (without replacement), and a single pseudo-word was randomly selected from each ending's list. For both Similar Probe and Dissimilar Probe trials (see Table 1), the test item matched one of the study items. For Similar Lure trials, the test item was a non-matching pseudo-word that came from the same vowel-based ending as

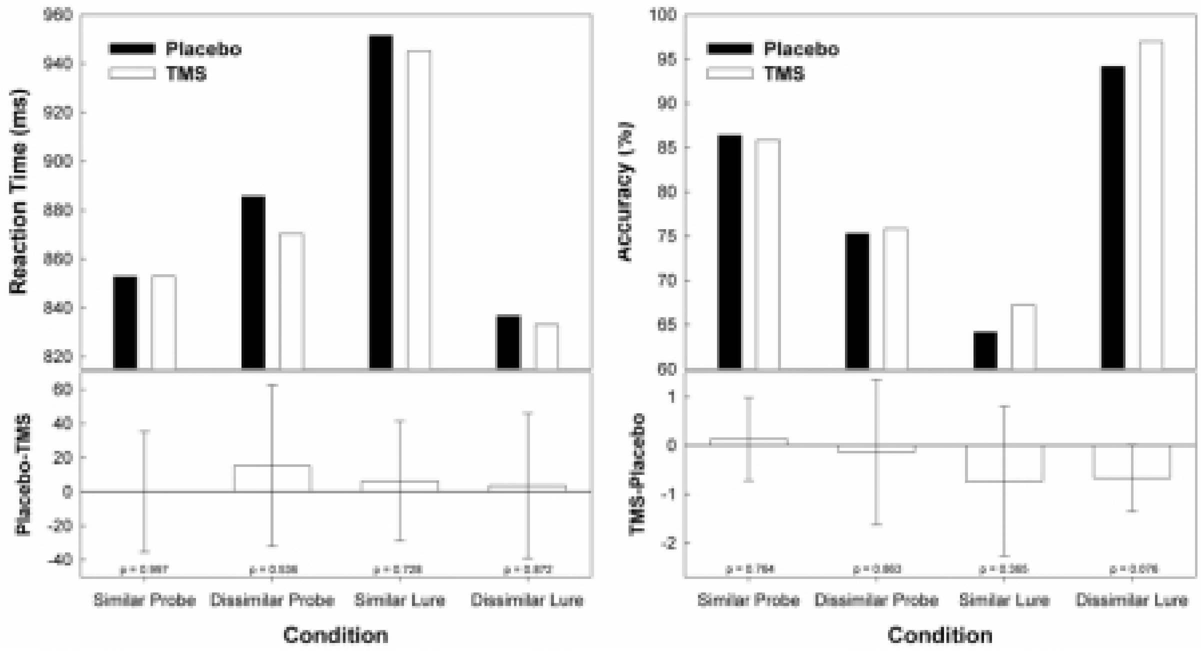

Fig. 4. Response time and accuracy plots for control stimulation, The left and right plots are analogous to the response time and accuracy plots in Fig. 3 for the control or parieto-occipital junction site.

did the study items. For Dissimilar Lure trials, the non-matching test item was randomly selected from a vowel-based ending that was different from all of the study items.

### 2.6. *TMS protocol*

Volume renderings of each subject's brain were created from the SPGR scan. Left motor cortex and LIP activations were localized using the Brainsight-Frameless system (Rogue Research Inc.) [11]. Stimulation magnitude for the phonological similarity task was set at 120% of a subject's motor threshold (as determined by the lowest intensity stimulation pulse required to produce a hand twitch from motor cortex stimulation). Three TMS pulses were applied to the LIP region at 1, 3 and 5 seconds from the end of the last stimulus during the 6 second rehearsal interval with a flat 70 mm double coil driven by a Magstim 220 Biphasic Stimulator. The TMS coil was oriented with the handle perpendicular to the central sulcus and pointing towards the back of the head [22]. Placebo stimulation was achieved by rotating the TMS coil 90 degrees tangential to the head. Stimuli were presented using E-Prime software (Psychology Software Tools, Inc.).

## 3. Results

Results indicated that following TMS stimulation to LIP, memory for phonologically similar stimuli was significantly faster ($p = 0.046$, two-tailed paired t-test) and marginally more accurate ($p = 0.066$, two-tailed paired t-test) than after placebo stimulation (Fig. 3). Specifically, TMS administration allowed subjects to reject phonologically similar lures more quickly and accurately relative to the placebo condition. No differences between TMS and placebo stimulation were observed for phonologically dissimilar stimuli, or for phonologically similar probes, indicating that the improved performance was not a non-specific effect attributable, for example, to heightened arousal due to scalp muscle twitching. A subset of these subjects ($N = 12$) also completed the behavioral task in a baseline condition with no TMS or placebo stimulation. There were no significant response time differences between the baseline condition and placebo stimulation (repeated measures analysis of variance, $F(1, 11) = 0.29$, NS) and no significant interaction between the two conditions ($F(3, 33) = 0.52$, NS), indicating that the auditory distraction produced by the click of placebo stimulation was not capable of reproducing the effects observed from TMS.

Table 1
Summary of trial types presented to each subject. Each trial condition is characterized by the type of study items presented, either phonologically similar or dissimilar, and by the type of test item presented, either probe (matching) or lure (non-matching). In the examples for each cell, study items appear in normal typeface, while test items are italicized

| | | Test Item | |
|---|---|---|---|
| | | Matching (Probe) | Non-Matching (Lure) |
| Phonological Characteristic of Study Items | Similar | Condition: Similar Probe<br>Ex: mell, rell, gell,. . . *gell* | Condition: Similar Lure<br>Ex: mell, rell, gell,. . . *vell* |
| | Dissimilar | Condition: Dissimilar Probe<br>Ex: shen, floy, stap,. . . *floy* | Condition: Dissimilar Lure<br>Ex: shen, floy, stap,. . . *krup* |

To ensure that these behavioral effects in similar lure trials accurately reflect targeted stimulation of the LIP lobule, and to investigate whether these results could be attributed to TMS stimulation of the brain in general or increased arousal as a result of scalp or neck muscle stimulation, an additional 15 subjects completed the experiment with TMS and placebo stimulation applied to a control region, the parieto-occipital junction at the midline (Fig. 4). Subjects in this cohort showed a positive PSE, but failed to demonstrate any difference in either response time (two-tailed paired t-test, NS) or accuracy (two-tailed paired t-test, NS) between TMS and placebo stimulation in any condition, confirming that focal TMS to the LIP region is sufficient to enhance phonological memory.

## 4. Discussion

Our results indicate that faster and more accurate performance occurred under the difficult Dissimilar Lure condition when TMS was administered to the LIP cortex, but not to the control region. The results of the present experiment may seem somewhat paradoxical in that TMS to the LIP cortex produced facilitation of VWM performance, and yet damage to this region typically results in disruption of VWM performance as seen, for example, by measures of decreased digit span. However, in addition to a general working memory impairment, a second consequence of LIP damage is nearly identical working memory recall for phonologically similar and dissimilar lists of items [3,30], a phenomenon that is not typically observed in patients with damage to other regions. The patient lesion studies support the conclusion that an intact phonological store is necessary for normal working memory capacity, and that a natural consequence of a phonologically-based short-term memory mechanism is its susceptibility to interference by similar-sounding items. Patients with LIP damage lose working memory capacity, but at the same time also lose the tendency for phonologically-based interference, and hence, show a reduced PSE.

With these conclusions from patient lesion studies in mind, a reasonable explanation for the results of the present experiment are that, first, the stimulation parameters for TMS were not optimum for disrupting the phonological store sufficiently to cause memory impairments. Since TMS in the present study was only applied during the rehearsal period, it is likely that the items were properly encoded and successfully transferred into the phonological loop (shown by the high accuracy rates). In the dynamic nature of the phonological loop, items in the phonological store are rapidly refreshed by the articulatory control system. Since TMS was applied at low frequency, it is reasonable to conceive that the refresh rate of the phonological store is faster than the duration of the TMS disruption, and thus memory for the items themselves was not degraded and could be successfully maintained by the action of the phonological loop despite TMS stimulation. The question then remains as to why, in concordance with results observed in LIP patients, TMS selectively reduced phonological interference during phonologically similar trials.

One plausible explanation stems from item-item associations that are formed within the LIP region. As items in memory are circulated through the phonological loop, the strength of the connection between adjacent items, or the item-item association, increases with each round of rehearsal. Associations between phonologically similar items have inherently greater strength relative to dissimilar items because of their related phonetic structure. These tight connections between phonologically similar items make it difficult to distinguish between individual items in memory, producing interference during retrieval that gives rise to the PSE. The item-item associations between phonologically dissimilar items, on the other hand, are much weaker, facilitating the retrieval of a specific single item when necessary. It is reasonable to believe that these associations are formed during the brief period when the items are resident in the phonological store. TMS, by disrupting the phonological store, may inhibit the formation of these item-item associations, thereby improving the ability to differentiate between phonologically similar items. Under this model, it is not surprising that TMS did not improve memory for phonologically dissimilar items because their item-item associations were already inherently weak.

The present study is the first to our knowledge to demonstrate the use of TMS to reproduce the neurobehavioral patterns of phonological memory performance observed in LIP-damaged patients. Several studies exploring working memory have reported impairments in task performance following TMS [15,16,24–26]. Most of these studies stimulated the dorsal lateral prefrontal cortex and used high frequency repetitive TMS. The only published study of TMS application specifically to the LIP region showed a non-significant trend towards higher error rates compared to stimulation of the contralateral mirror control site in a similar VWM paradigm [16]. This finding is consistent with results from the phonologically dissimilar conditions in the present study which show no behavioral differences between TMS and placebo stimulation. Only by examining the phonologically similar conditions do we observe an improvement in memory performance and gain further evidence for the role of the LIP as a neuroanatomical correlate of the short-term phonological store.

The current investigation demonstrates the value of TMS as a research tool, allowing us to test hypotheses about how cognitive information is processed in the brain that extend beyond what is approachable through neuroimaging. The capability of TMS to create transient changes in memory performance and neurocognitive profiles mirroring those of patients with cortical lesions is a substantial advancement for neuroscience and will yield novel information about brain-behavior relationships.

## Acknowledgements

We would like to thank Dr. Gary Glover for continued fMRI support, Dr. Annabel Chen for TMS support, and Jenea Boshart for project coordination. Supported by NIMH (MH60234) and Stanford Medical Scientist Training Program.
